# CK1δ as a potential therapeutic target to treat bladder cancer

**DOI:** 10.18632/aging.102966

**Published:** 2020-04-13

**Authors:** Yu-Chen Lin, Mei-Chuan Chen, Tsung-Han Hsieh, Jing-Ping Liou, Chun-Han Chen

**Affiliations:** 1 Department of Pharmacology, School of Medicine, College of Medicine, Taipei Medical University, Taipei, Taiwan; 2 Ph.D. Program in Clinical Drug Development of Herbal Medicine, College of Pharmacy, Taipei Medical University, Taipei, Taiwan; 3 Traditional Herbal Medicine Research Center of Taipei Medical University Hospital, Taipei, Taiwan; 4 Joint Biobank, Office of Human Research, Taipei Medical University, Taipei, Taiwan; 5 School of Pharmacy, College of Pharmacy, Taipei Medical University, Taipei, Taiwan; 6 Cell Physiology and Molecular Image Research Center, Wan Fang Hospital, Taipei Medical University, Taipei, Taiwan; 7 TMU Research Center of Cancer Translational Medicine, Taipei Medical University, Taipei, Taiwan

**Keywords:** bladder cancer, CK1δ, apoptosis, necroptosis, migration

## Abstract

Bladder cancer is the second most common genitourinary malignancy in the world. However, only immune-checkpoint inhibitors and erdafitinib are available to treat advanced bladder cancer. Our previous study reported that 4-((4-(4-ethylpiperazin-1-yl) phenyl)amino)-N-(3,4,5-trichlorophenyl)-7H-pyrrolo-[2, 3-d]pyrimidine-7-carboxamide hydrochloride (13i HCl) is a potent CK1δ inhibitor showing significant anti-bladder cancer activity. In this study, we elucidated the pharmacological mechanisms underlying 13i HCl’s inhibition of human bladder cancer. Our results demonstrate that expression of the *CSNK1D* gene, which codes for CK1δ, is upregulated in superficial and infiltrating bladder cancer patients in two independent datasets. CK1δ knockdown decreased β-catenin expression in bladder cancer cells and inhibited their growth. Additionally, 13i HCl suppressed bladder cancer cell proliferation and increased apoptosis. We also observed that inhibition of CK1δ using 13i HCl or PF-670462 triggers necroptosis in bladder cancer cells. Finally, 13i HCl inhibited bladder cancer cell migration and reversed their mesenchymal characteristics. These findings suggest further development of 13i HCl as a potential therapeutic agent to treat bladder cancer is warranted.

## INTRODUCTION

Bladder cancer is the second most common genitourinary malignancy in the world, with an estimated 549,393 new cases and 199,922 deaths in 2018 [1]. In the United States, an estimated 80,470 people will be diagnosed with this disease and 17,670 people are expected to have died from it in 2019 [2]. Statistically, 70% of patients are newly diagnosed with non-muscle invasive bladder cancers (NMIBC), which have a five-year survival rate of ~90%. However, NMIBCs have a high recurrence rate and a high probability of progressing toward muscle invasive bladder cancers (MIBCs) [3], with a dramatically reduced five-year survival rate once the disease becomes metastatic [4], since treatment for metastatic bladder cancer has seen little progress in decades [3]. For MIBC, the standard of care is radical cystectomy with platinum-based chemotherapy. The most active regimens are methotrexate, vinblastine, doxorubicin, and cisplatin (MVAC), dose-dense MVAC, and gemcitabine plus cisplatin [5, 6]. Patients who received platinum-based chemotherapy have an overall survival rate of 9-15 months. Still, the median survival is reduced to 5 to 7 months in patients with resistance to platinum-based chemotherapy [7]. Immunotherapy by checkpoint inhibitors is the second-line of therapy for patients who fail to respond to first-line chemotherapy [8]. While erdafitinib, a pan-FGFR inhibitor, has been recently approved as a monotherapy option for patients with locally-advanced or metastatic urothelial cancer [9], caring for bladder cancer patients remains a huge social problem due to the high economic burden from end-of-life care, high recurrence rate of NMIBCs, and lack of effective treatments [10]. Accordingly, there is an unmet need to develop novel therapeutic agents for advanced bladder cancer patients.

CK1δ and CK1ε are two structure-related serine/threonine kinases with high homology in their kinase (98%) and C-terminal regulatory domains (53%) [11]. Several of their common substrates are involved in oncogenic signaling, such as Wnt (APC, β-catenin, NFATC3), p53 (TP53, MDM2), and death-receptor signaling (FADD, BID) [12], triggering gene transcription [13]. Due to the structural similarity and functional overlap, the contributions of CK1δ and CK1ε to the progression of human cancers remain elusive. Rosenberg et al. reported that CK1δ is widely overexpressed within a subset of breast tumors across all major classes, while CK1ε overexpression is restricted to the basal-like subclass by analyzing the transcription level of CK1 isoforms in datasets from the Cancer Genome Atlas (TCGA). Meanwhile, copy number gains of the *CSNK1D* locus were found in 36% of breast tumors, with higher frequencies in the basal-like and luminal B subtypes. The authors also revealed that CK1δ is a driver of Wnt/β-catenin activation, a molecular phenotype known to associate with poor prognosis in breast cancer patients [14, 15]. Importantly, either APC mutations or nuclear β-catenin accumulation are associated with poor outcome in patients with invasive bladder cancer [16]. Evidence from the microarray database of tumor cell lines and tissue samples indicated that CK1δ is overexpressed in many types of malignancy, including bladder cancer [12]. A TCGA dataset also showed that the copy number of *CSNK1D*, the gene that codes for CK1δ, is amplified in ~50% of bladder tumors, which correlated with enhanced CK1δ expression [14]. In addition, there was a large overlap between the CK1δ gene signature and Wnt signaling genes in bladder cancer [14, 15]. Together, the evidence suggests that CK1δ inhibition may be a promising strategy to treat human bladder cancer.

We previously identified 7*H*-pyrrolo-[2,3-*d*]pyrimidine derivatives as novel anticancer agents with potent anti-CK1δ activity [17]. Importantly, 4-((4-(4-ethylpiperazin-1-yl)phenyl)amino)-N-(3,4,5-trichlorophenyl)-7H-pyrrolo-[2, 3-d]pyrimidine-7-carboxamide hydrochloride (13i HCl) exhibits stronger anticancer activities than known CK1δ/ε inhibitors (PF-4800567, D4476, PF-670462) in human bladder and ovarian cancer cells. The inhibition of the CK1 δ/β-catenin pathway partly contributes to 13i HCl-mediated cell death. In the present study, we further elucidated the action mechanisms of 13i HCl in human bladder cancers. Our results here demonstrated that *CSNK1D* was upregulated in superficial and infiltrating bladder cancer patients from two independent datasets. Furthermore, compound 13i HCl suppresses proliferation and increases apoptosis in bladder cancer cells. For the first time, our data suggested that inhibition of CK1δ activates necroptosis in bladder cancer cells. Finally, 13i HCl inhibits migration of bladder cancer cells and reverses their mesenchymal characteristics. In conclusion, our findings describe the pharmacological mechanisms of compound 13i HCl in a preclinical setting, highlighting it as a potential therapeutic agent to treat bladder cancer.

## RESULTS

### CK1δ is crucial to the growth of bladder cancer cells

**Figure 1:**
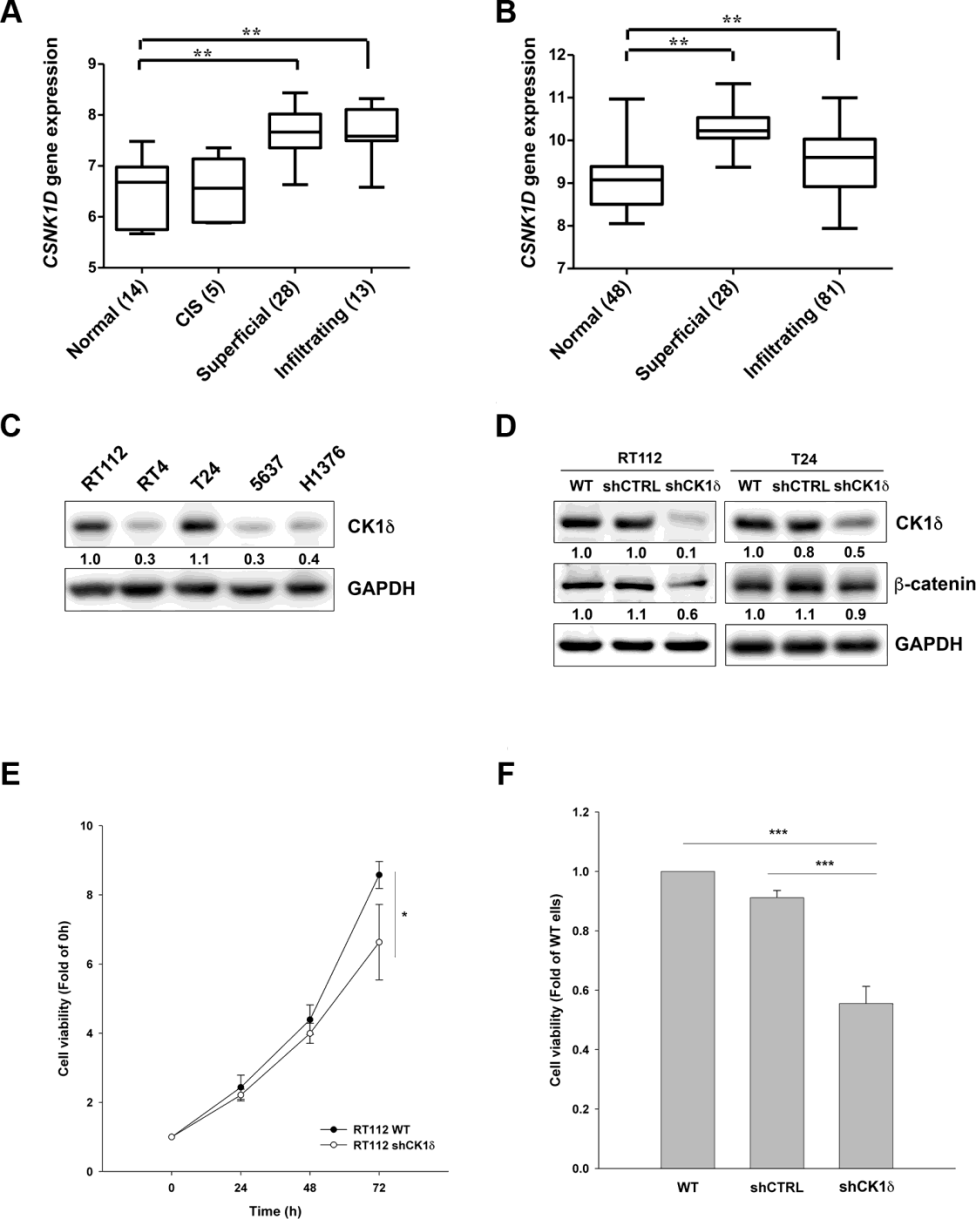
**CK1δ promotes growth of bladder cancer cells.** (**A**, **B**) Gene expression levels of *CSNK1D* in tissue samples of normal, carcinoma *in situ* (CIS), superficial and infiltrating bladder cancer patients obtained from Dyrskjot bladder dataset (**A**) or Sanchez-Carbayo bladder dataset (**B**). **P<0.01 compared to normal group. (**C**) Protein levels of CK1δ in different bladder cancer cell lines analyzed by Western blotting. (**D**) Control shRNA (shCTRL) or shCK1δ plasmids were transduced into RT112 and T24 cells by lentivirus and the cells were subjected to Western blotting with the indicated antibodies. (**E**, **F**) CK1δ knockdown decreases the viability of RT112 (**E**) and T24 (**F**) cells at 72 h by MTT assay. Date are represented as mean ± S.D. *P<0.05, ***P<0.001 compared to wild type (WT) cells (n=3).

To explore the relationship between CK1δ levels and bladder cancer progression in a clinical setting, we analyzed two independent microarray datasets of mRNA levels in normal tissues and patient samples. The results demonstrated that the gene expression of *CSNK1D* was upregulated in superficial and infiltrating bladder cancer patients (Figure 1A, 1B). We also examined CK1δ protein levels in different bladder cancer cell lines, and found that RT112 and T24 express the highest levels of CK1δ (Figure 1C). We therefore chose these two cell lines for subsequent experiments. To evaluate the contribution of CK1δ to cell growth, we stably knocked down *CSNK1D* by lentiviral transduction. The data suggested that CK1δ levels and those of its downstream target, β-catenin, were decreased in RT112 and T24 cells (Figure 1D). Meanwhile, viability decreased for RT112 and T24 cells at 72 h (Figure 1E, 1F). Together, the data suggest that CK1δ contributes to cell growth in bladder cancer cells.

### Compound 13i HCl exhibits anti-proliferative activity in bladder cancer cells

**Figure 2:**
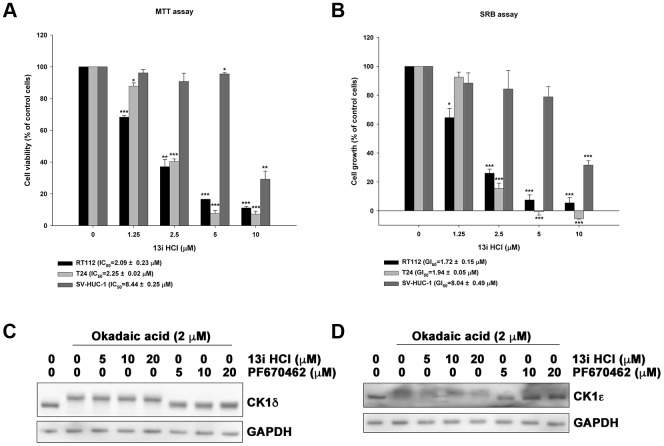
**Compound 13i HCl exhibits anti-proliferative activity in bladder cancer cells.** (**A**, **B**) RT112, T24 and SV-HUC-1 cells were exposed to the indicated concentrations of 13i HCl for 48 h and subjected to MTT assay (**A**) or SRB assay (**B**) to analyze cell viability and proliferation, respectively. Data are represented as mean ± S.D. (n=3) *P<0.05, **P<0.01, ***P<0.001 compared to control cells. (**C**, **D**) RT112 cells were treated with the indicated concentrations of 13i HCl and PF670462 in the presence of okadaic acid (2 μM) for 1 h and subjected to Western blotting by using CK1δ (**C**) and CK1ε (**D**) antibodies.

We previously reported 7*H*-pyrrolo-[2,3-*d*]pyrimidine derivatives as novel anticancer agents with potent anti-CK1δ activity [17]. Among them, 13i HCl is the most potent against human RT-112 bladder cancer cells. In the current study, we evaluated the anti-proliferative activity of 13i HCl using MTT and SRB assay in two bladder cancer cell lines which express the highest levels of CK1δ. The data revealed that 13i HCl decreased the viability of RT112 and T24 cells in a concentration-dependent manner (Figure 2A) and inhibited their proliferation (Figure 2B). Notably, 13i HCl displayed weaker effects on the viability and proliferation of normal uroepithelial SV-HUC-1 cells (Figure 2A, 2B). As CK1δ and CK1ε are highly similar in their kinase domain, we further used autophosphorylation assay to examine the inhibitory activity of 13i HCl on CK1δ and CK1ε in RT112 cells [18]. CK1δ and CK1ε usually autophosphorylate their carboxyl-terminus regulatory domain, and the reaction is reversed by phosphatases which are sensitive to the inhibitor, okadaic acid, resulting in a rapid electrophoretic mobility shifts in western blots. Our data revealed that compound 13i HCl inhibited the autophosphorylation of CK1δ and CK1ε in a concentration-dependent manner, resembling the known dual CK1δ/ε inhibitor PF670462 (Figure 2C, 2D). In summary, 13i HCl exhibits potent anti-cancer activity in bladder cancer cell lines.

### Effects of compound 13i HCl on cell cycle progression and apoptotic pathways in bladder cancer cells

**Figure 3:**
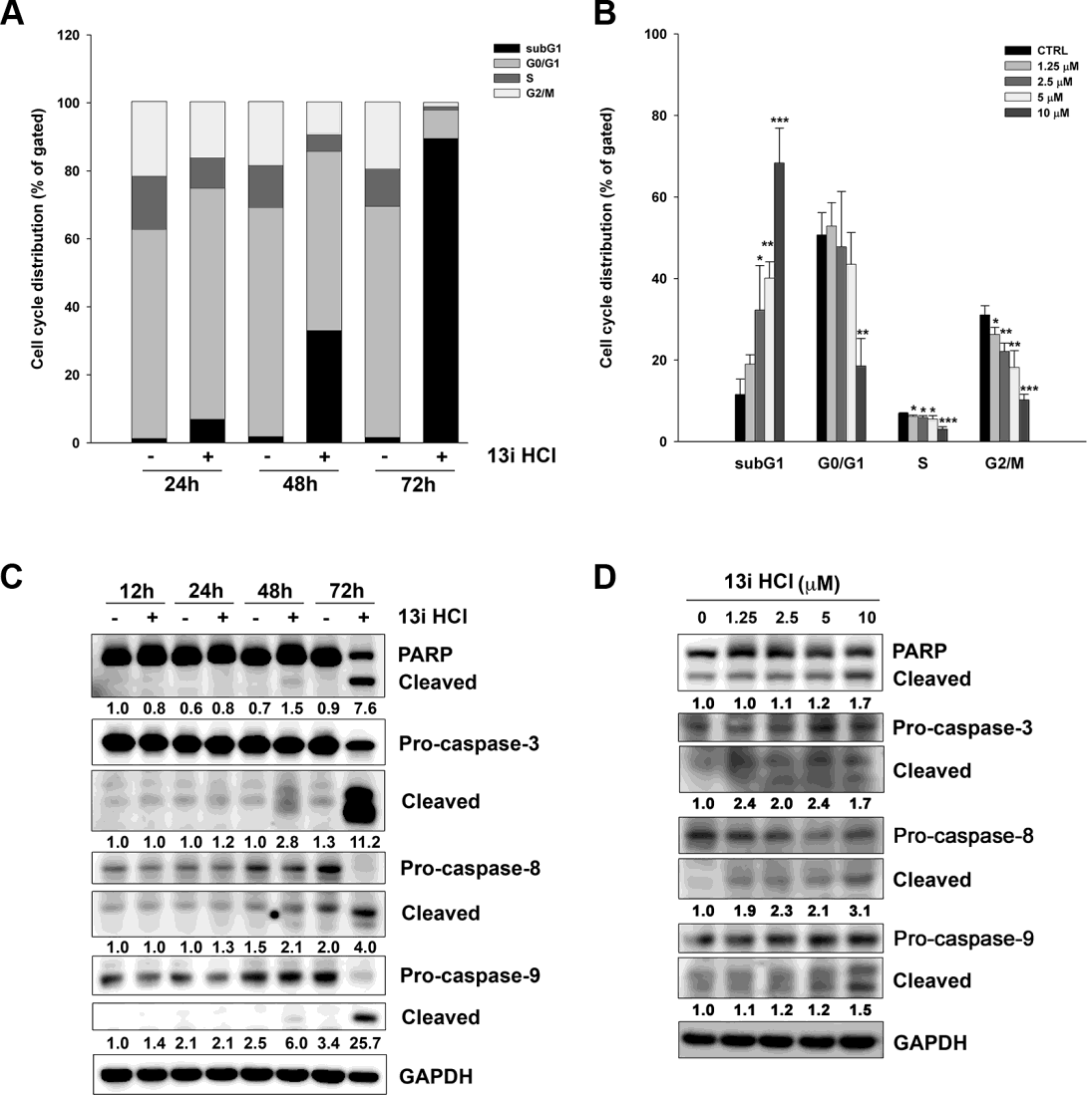
**Compound 13i HCl induces apoptosis in RT112 cells.** (**A**) RT112 cells were exposed to the indicated concentrations of 13i HCl for 72 h and subjected to cell cycle analysis. Data are represented as mean ± S.D. *P<0.05, **P<0.01, ***P<0.001 compared to control cells (n=3). (**B**) RT112 cells were treated with 13i HCl (10 μM) for the indicated times and cell cycle distribution was measured by PI staining and flow cytometry (n=2). (**C**, **D**) RT112 cells were exposed to the indicated concentrations of 13i HCl for 72 h (**C**) or 13i HCl (10 μM) for different times (**D**) and subjected to Western blotting with the indicated antibodies.

To elucidate the mechanism underlying 13i HCl-induced cell death, we first examined 13i HCl’s effect on cell cycle progression by propidium iodide (PI) staining and flow cytometry. The data revealed that 13i HCl increased the population of sub-G1 cells in RT112 cells in a time- and concentration-dependent manner (Figure 3A, 3B). Accordingly, we further examined the regulatory proteins of apoptosis by western blotting. Compound 13i HCl activated the cleavage of caspase-3, -8, -9 as well as PARP in a time- and concentration-dependent manner in RT112 cells (Figure 3C, 3D). We also confirmed that 13i HCl increased the number of cells at the sub-G1 phase and apoptosis in T24 cells in a concentration-dependent manner (Supplementary Figure 1). Collectively, these data suggest that 13i HCl increases apoptosis in bladder cancer cells.

### Inhibition of CK1δ activates necroptosis in bladder cancer cells

**Figure 4:**
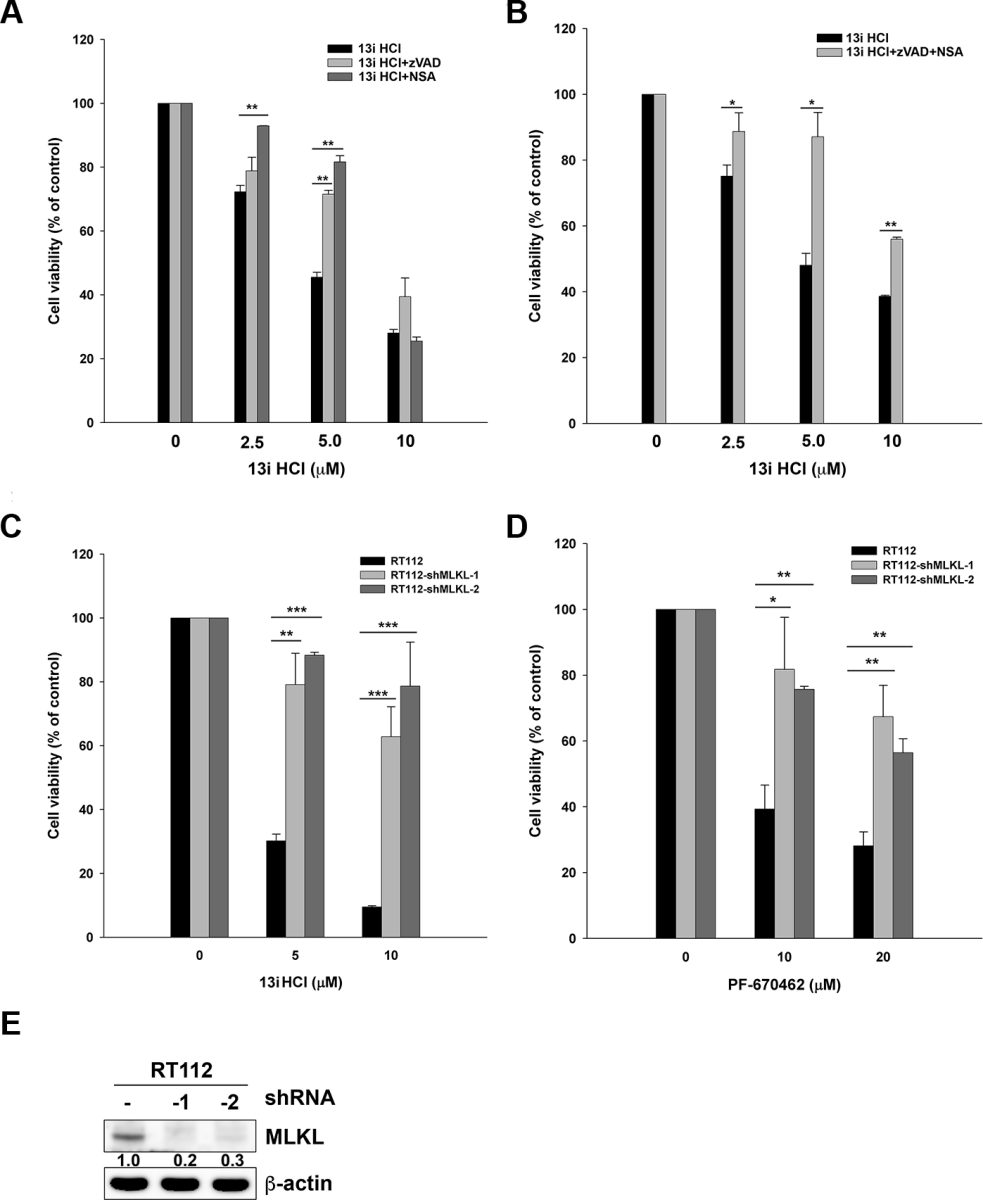
**Inhibition of CK1δ activates necroptosis in bladder cancer cells.** (**A**) RT112 cells were treated with the indicated concentrations of 13i HCl in the presence or absence of zVAD (20 μM) or NSA (10 μM) for 48 h and subjected to MTT assay. Data are represented as mean ± S.D. **P<0.01 compared to the group of 13i HCl alone (n=3). (**B**) RT112 cells were treated with the indicated concentrations of 13i HCl in the presence or absence of zVAD (20 μM) plus NSA (10 μM) for 48 h and subjected to MTT assay. Data are represented as mean ± S.D. *P<0.05, **P<0.01 compared to the group of 13i HCl alone (n=3). (**C**, **D**) RT112 and MLKL stable knocked-down clone (shMLKL-1, shMLKL-2) cells were treated with the indicated concentrations of 13i HCl (**C**) or PF-670462 (**D**) for 72 h and subjected to MTT assay. Data are represented as mean ± S.D. *P<0.05, **P<0.01, ***P<0.001compared to the group of 13i HCl or PF-670462 alone (n=3). (**E**) The knockdown efficiency of MLKL in RT112 cells was examined by Western blotting.

From the above findings, we observed that the protein levels of GAPDH were decreased under high concentrations of 13i HCl (Figure 3C, 3D). We hypothesized that compound 13i HCl increased membrane permeability in bladder cancer cells. Both apoptosis and necroptosis are classified as programmed cell death under drug-induced stress [19, 20]. We therefore examined the effect of apoptosis- and necroptosis-inhibitors on 13i HCl-induced cell death. The results showed that a pan-caspase inhibitor, Z-VAD-FMK, rescued cell death in the presence of 5 μM 13i HCl. Necrosulfonamide (NSA), a necroptosis inhibitor, reversed the cell death induced by 2.5 and 5 μM 13i HCl (Figure 4A). However, neither of these drugs reversed cell death at 10 μM. Because inhibition of caspases by Z-VAD-FMK might further increase the number of cells undergoing necroptosis, we therefore combined Z-VAD-FMK and NSA to examine their compounded effect on 13i HCl-induced cell death. The data showed that Z-VAD-FMK together with NSA reversed cell death induced by 2.5 to 10 μM of 13i HCl (Figure 4B). To further confirm the contribution of necroptosis in 13i HCl-induced cell death, we stably knocked down mixed lineage kinase domain-like protein (MLKL), the key signaling molecule in necroptosis. Surprisingly, MLKL-knockdown rescued 13i HCl-induced cell death (Figure 4C). Meanwhile, cell death induced by PF-670462, a specific CK1δ/ε inhibitor, was also rescued by MLKL-knockdown (Figure 4D). The knockdown efficiency was confirmed by western blotting (Figure 4E). Together, the data suggest that inhibition of CK1δ triggers not only apoptosis, but also necroptosis in bladder cancer cells.

### Compound 13i HCl triggers the phosphorylation of MLKL and ROS production in bladder cancer cells

**Figure 5:**
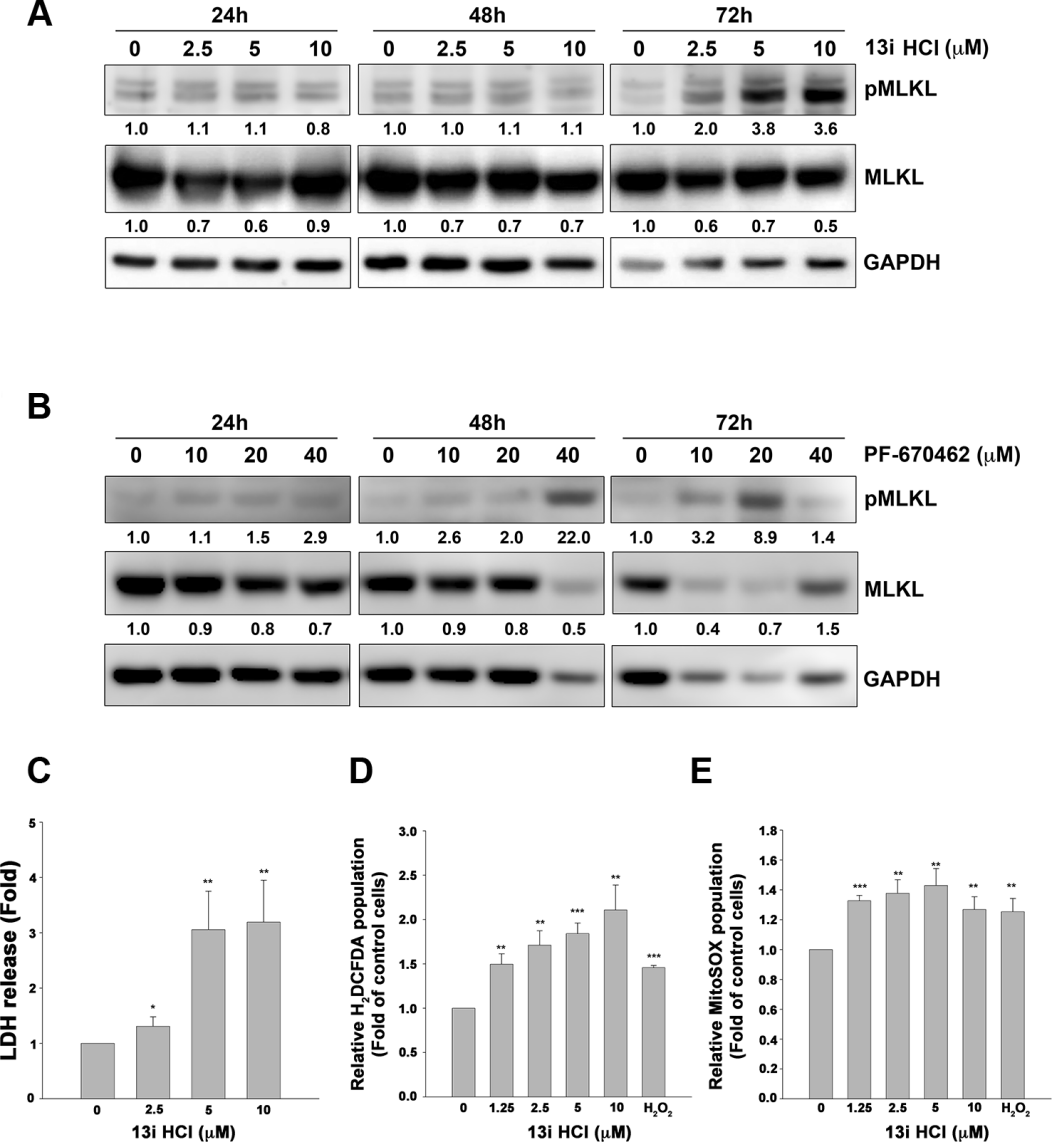
**Compound 13i HCl increases MLKL phosphorylation and ROS production in bladder cancer cells.** (**A**, **B**) RT112 cells were treated with the indicated concentrations of 13i HCl (**A**) or PF-670462 (**B**) for 24 h, 48 h and 72 h. The cells were subjected to Western blotting with the indicated antibodies. (**C**) RT112 cells were exposed to 13i HCl for 72 h and LDH levels in the supernatants were determined. (**D**, **E**) RT112 cells were exposed to the indicated concentrations of 13i HCl for 72 h or H_2_O_2_ (10 μM) for 1 h. The cells were stained with H_2_DCFDA (**D**) or MitoSOX (**E**) and subjected to flow cytometry. Data are represented as mean ± S.D. *P<0.05, **P<0.01, ***P<0.001 compared to control cells (n=3).

To further test whether compound 13i HCl increases necroptosis in bladder cancer cells, we evaluated the marker of necroptosis, phosphorylated MLKL (pMLKL) by western blotting. The data showed that 13i HCl increased the phosphorylation of MLKL in a concentration-dependent manner at 72 h (Figure 5A). The same phenomenon was observed in RT112 cells treated with a known CK1δ/ε inhibitor, PF-670462 (Figure 5B). Meanwhile, 13i HCl increased extracellular LDH levels in a concentration-dependent manner (Figure 5C). It is known that reactive oxygen species (ROS) contribute to triggering necroptosis and apoptosis [21, 22]. Mitochondrial ROS reportedly promote RIP1 autophosphorylation, which is crucial for the recruitment of RIP3 in the necroptosome [23]. We therefore examined intracellular and mitochondrial ROS levels by using H_2_DCFDA and MitoSOX staining, respectively. Here we show that 13i HCl increased intracellular and mitochondrial ROS levels in RT112 cells (Figure 5D, 5E). Together, the data suggested that 13i HCl triggers necroptosis in bladder cancer cells.

### Effects of 13i HCl on migratory activity in bladder cancer cells

**Figure 6:**
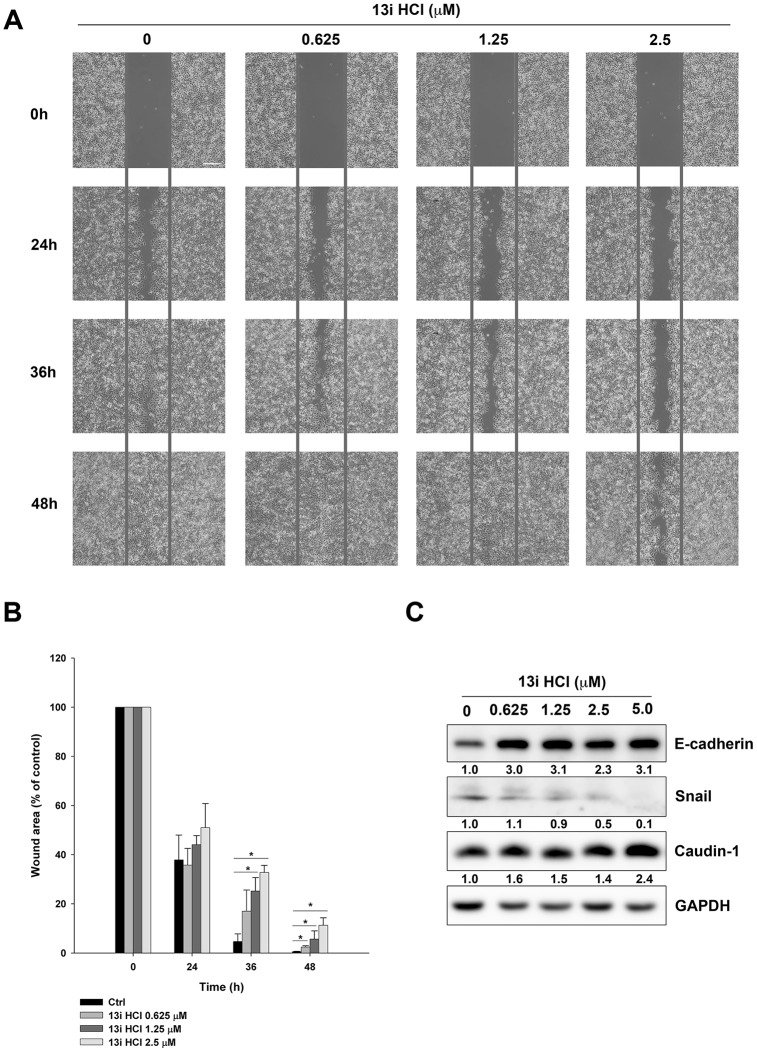
**Effects of 13i HCl on migratory activity of bladder cancer cells.** (**A**) RT112 cells were seeded into a 2-well insert on 6-well plate and allowed to attach overnight. The wound was created by removing the insert, and the cells were treated with or without 13i HCl and the images were captured at indicated times by EVOS XL Core Cell Imaging System (Thermo Scientific). Scale bar = 100 μm. (**B**) Quantification of wound healing assay. Data are represented as mean ± S.D. (n=2) *P<0.05 compared to control cells. (**C**) RT112 cells were exposed to indicated concentrations of 13i HCl for 72 h and subjected to Western blotting for the detection of EMT markers.

Our previous study demonstrated that compound 13i HCl displays inhibitory activity against CK1δ [17]. As CK1δ regulates several mediators of cancer metastasis, such as wnt/β-catenin and metastasis suppressor 1 (MTSS1) [12, 24], we examined the effects of 13i HCl on migration by wound-healing assay. Our data showed that 13i HCl inhibited the migratory activity of RT112 and T24 cells in a concentration-dependent manner (Figure 6A, 6B; Supplementary Figure 2A, 2B). Additionally, knocking-down CK1δ also decreased wound-healing in RT112 and T24 cells (Supplementary Figure 3A, 3B). Meanwhile, 13i HCl reversed the expression of epithelial-mesenchymal transition (EMT) markers, such as the increase in E-cadherin, and decrease in snail expression in RT112 cells (Figure 6C). Therefore, the data suggested that 13i HCl decreases migration and reversed EMT marker levels in bladder cancer cells.

## DISCUSSION

Bladder cancer is the second most common genitourinary malignancy worldwide [1]. Approximately 30% of patients are first diagnosed as MIBC with a five-year survival rate of 50%, and that drops to only 6% once it progresses to the metastatic stage [4]. For decades, platinum-based chemotherapy has been the standard-of-care for advanced bladder cancer, but patients are often non-responsive or develop of resistance [3]. Hence, there is an unmet need to develop novel therapeutic agents to treat bladder cancer. CK1δ is a serine/threonine-protein kinase that regulates many cellular processes implicated in cancer, including Wnt/β-catenin signaling, apoptosis, DNA damage response and circadian rhythms [12]. Copy number amplification was observed in 50% of bladder tumors, which correlated with CK1δ overexpression [12, 14]. However, the anticancer activity and pharmacological mechanisms of CK1δ inhibitors on bladder cancer cells remain elusive. In the current study, we reported that mRNA levels of *CSNK1D*, the coding gene of CK1δ, are upregulated in both of superficial and invasive bladder cancers by analyzing two independent datasets (Figure 1A, 1B). Meanwhile, CK1δ is crucial to the growth and migration of bladder cancer cells (Figure 1D, 1F; Figure 6B). We also demonstrated that compound 13i HCl, a derivative of 7*H*-pyrrolo-[2,3-*d*]pyrimidines with CK1δ-inhibitory activity, inhibited proliferation and migration, and induced apoptosis in bladder cancer cells. Importantly, we describe for the first time that inhibition of CK1δ by 13i HCl or PF-670462 triggers necroptosis in bladder cancer cells. These results suggest that CK1δ could be a valuable therapeutic target to treat advanced bladder cancer, and that further development of compound 13i HCl is warranted to improve patient outcomes.

Several small molecules have shown Ck1δ-inhibitory activity, such as CKI-7, D4476, IC261, ®-DRF053, Bischof-524, and PF-670462 [25], but all present various problems that limit their application to bladder cancer therapy [26–31]. Recently, a series of purine scaffold inhibitors were discovered with IC_50_ values in the low nanomolar range, such as SR-1277, SR-2890, and SR-3029 [31]. Rosenberg et al. successfully demonstrated that the CK1δ/ε dual inhibitor SR-3029 inhibits growth in CK1δ-high breast cancer cells and several tumor xenografts in mice [14]. We also observed that compound 13i HCl inhibits both CK1δ and CK1ε isoforms as evidenced by autophosphorylation assays (Figure 2C, 2D). Currently, the challenge of using CK1 inhibitors to treat bladder cancer is the lack of selectivity between CK1δ and CK1ε, which may result from the high similarity in their kinase domain. Hence, drug design should focus on their highly variable C-terminal domains or the inhibition of auto-phosphorylation. The discovery of selective CK1δ- or CK1ε-specific inhibitors will provide new therapeutic possibilities for personalized medicine.

Although CK1δ inhibitors have shown promising anticancer activity in several studies, the molecular mechanisms underlying drug-mediated cell death are still unclear. In the current study, we observed that inhibition of CK1δ activity by compound 13i HCl triggers apoptotic and necroptotic cell death in bladder cancer cells. We also provided evidence that 13i HCl treatment or CK1δ knockdown inhibits RT112 cell migration (Figure 6A, 6B). CK1δ reportedly activates Wnt/β-catenin in breast cancer [14, 15] and CK1δ silencing reduces the migration and invasion of triple-negative breast cancer cells [32]. Importantly, either APC mutations or nuclear β-catenin accumulation are associated with poor outcome in patients with invasive bladder cancer [16]. In a previous study, we found that forced expression of β-catenin rescued cytotoxicity induced by 13i HCl in RT112 cells [17]. Therefore, targeting the CK1δ/β-catenin pathway is an attractive strategy to treat Wnt-driven bladder cancer. On the other hand, CK1δ has emerged as a potential target for other diseases. CK1δ inhibitors have been proposed to inhibit the phosphorylation of TDP-43, a pathological hallmark of central nervous system (CNS) diseases, such as amyotrophic lateral sclerosis (ALS) and frontotemporal dementia [33, 34]. CK1δ is also a potential target for Parkinson’s disease and pulmonary fibrosis [35, 36]. These observations further support the development of compound 13i HCl as a potential therapeutic target for other diseases.

Apoptosis, necroptosis and autophagic cell death are three main types of programmed cell death (PCD) induced by anticancer agents [19]. Apoptosis is the most studied PCD, characterized by cytoplasmic shrinkage, chromatin condensation, nuclear condensation, and membrane blebbing. The activation of caspases is a feature of apoptosis, accompanied by DNA/protein breakdown, and mitochondrial outer membrane permeabilization [37]. Necroptosis is a caspase-independent PCD, a form of necrotic death executed by receptor-interacting protein 1 (RIP1), RIP3, and Mixed Lineage Kinase Domain-Like (MLKL) protein [38]. Since resistance to apoptosis is one of the hallmarks of cancer, necroptosis-based cancer therapy has been proposed as a novel strategy for antitumor treatment [39]. Necroptosis is a unique cell-killing mechanism in response to severe stress or impaired apoptosis, which can be triggered by inflammatory cytokines, chemotherapeutic agents, and natural compounds [40]. Meanwhile, necroptotic tumor cells initiate adaptive immune responses by releasing damage-associated molecular patterns (DAMPs) into the microenvironment, activating dendritic and cytotoxic T cells that may suppress tumor progression [41]. For the first time, we observed that inhibition of CK1δ activity by compound 13i HCl or PF-670462 triggers necroptosis in bladder cancer cells. Knocking down of MLKL rescued cytotoxicity induced by 13i HCl or PF-670462 in RT112 cells (Figure 4C, 4D). However, the mechanisms by which CK1δ regulates necroptosis remain elusive. From the literature, CK1δ is able to phosphorylate T362 in the catalytic domain of protein phosphatase 5 (PP5), enhancing its phosphatase activity [42]. A crucial target of PP5 is Cdc37, a cochaperone of the Hsp90 complex, regulating the activation of protein kinase clients by Hsp90-Cdc37 [43, 44]. Interestingly, RIPK3 and MLKL, two important regulators in necrosomes, are clients of Hsp90-Cdc37 [45, 46]. Therefore, it would be useful to elucidate any potential linkages between CK1δ inhibition and of necroptosis induction in future studies.

## MATERIALS AND METHODS

### Cell culture, antibodies, and reagents

RT112, RT4, 5637 cells were cultured in RPMI 1640; T24 cells were cultured in McCoy's 5a, H1376 cells were cultured in EMEM, and SV-HUC-1 were cultured in F-12K, supplemented with 10% (v/v) FBS and 1% (v/v) antibiotic-antimycotic solution (Thermo Fisher Scientific, Waltham, MA, USA) at 37 °C in a humidified incubator containing 5% CO_2_. 4-((4-(4-ethylpiperazin-1-yl)phenyl)amino)-N-(3,4,5-trichlorophenyl)-7H-pyrrolo-[2, 3-d]pyrimidine-7-carboxamide hydrochloride (13i HCl) was synthesized by Dr. Jing-Ping Liou, as previously described [17]. PF-670462, okadaic acid, necrosulfonamide (NSA), and z-VAD-FMK were purchased from Cayman Chemical (Ann Arbor, MI, USA). 3-(4,5-Dimethylthiazol-2-yl)-2,5-diphenyltetrazolium bromide (MTT) and Sulforhodamine B (SRB) were obtained from Sigma Chemical Corp (St. Louis, MO, USA). Antibodies against various proteins were obtained from the following sources: caspase-3 from Novus biologicals (Littleton, CO, USA); CK1δ, pMLKL from Abcam (Cambridge, MA, USA); β-catenin from Santa Cruz Biotechnology (Santa Cruz, CA, USA); PARP, caspase-8, caspase-9 from Cell Signaling Technology (Danvers, MA, USA), and β-actin, MLKL, GAPDH from Genetex (Irvine, CA, USA).

### Cell viability, proliferation and lactate dehydrogenase (LDH) assays

Cells were seeded in 96-well plates and exposed to indicated compounds for 48 or 72 h. Cell viability was examined by MTT assay as described previously [47]. Cell proliferation was measured with the SRB assay as described previously [48]. For LDH assay, cells were seeded in 96-well plates and treated with drugs at the indicated concentrations for 72 h. LDH levels in culture supernatants were analyzed by using the CytoTox 96 Non-Radioactive Cytotoxicity Assay Kit (Promega; Madison, WI, USA) according to the manufacturer's protocol.

### Cell cycle analysis

Cells were seeded in 6-well plates, and exposed to indicated compounds for 24 to 72 h. The cells were collected by trypsinization, washed one time by PBS, and fixed with ethanol (70%) at -20 °C overnight. The cells were pelleted by centrifugation, and incubated in 0.1 mL of phosphate-citric acid buffer (0.2 M NaHPO_4_, 0.1 M citric acid, and pH 7.8) for 15 min at room temperature, and then resuspended in propidium iodide staining buffer containing Triton X-100 (0.1%, v/v), RNase A (100 μg/mL), and propidium iodide (80 μg/mL) for 30 min in the dark. Cell cycle distribution was analyzed by flow cytometry with CellQuest software (Becton Dickinson, Mountain View, CA, USA) following previously published methods [17].

### Intracellular and mitochondrial ROS analysis

Cells were seeded in 6-well plates, exposed to DMSO or indicated compounds for 72 h, and harvested by trypsinization. For total cell ROS analysis, cells were stained with 0.1 μM H_2_DCFDA (Biotium, Fremont, CA, USA) at 37 °C for 20 min. For mitochondrial ROS analysis, cells were stained with 5 μM MitoSOX Red (Thermo Fisher Scientific, Waltham, MA, USA) at 37 °C for 10 min. After washing with PBS three times, cells were subjected to ROS detection via flow cytometry with CellQuest software according to the manufacturer's instructions (Becton Dickinson, Mountain View, CA, USA), as previously described [47].

### Western blot analysis and lentiviral expression system

The cells were seeded in 6-well plates or 60 mm dishes and exposed to different compounds for the indicated times. After treatment, equal amounts of protein were separated via SDS–PAGE, transferred to PVDF membrane and immunoblotted with specific antibodies, as described previously [47]. Lentiviral particles containing shRNA plasmids of shCSNK1D (TRCN0000001552) and shMLKL (TRCN0000194846 and TRCN0000196741) were purchased from the National RNAi Core Facility (Academia Sinica, Taiwan). Stable cell lines were selected by the treatment of puromycin (InvivoGen; San Diego, CA, USA).

### Cell migration assay

Cells were seeded into a 2-well insert (Ibidi, Munich, Germany) on a 6-well plate and allowed to attach overnight. A wound was created by removing the insert, and the cell-free gap was around 500 μm. Cells were allowed to migrate in the presence or absence of drugs, and images were captured at the indicated times by EVOS XL Core Cell Imaging System (Thermo Scientific).

### Microarray datasets analysis

Dyrskjot et al. published a dataset in which 14 normal bladder, 5 carcinoma *in situ* (CIS), 28 superficial bladder cancer, and 13 invasive bladder cancer samples were analyzed using Affymetrix U133A microarrays [49]. Array data were obtained from the NCBI Gene expression omnibus (GEO; http://www.ncbi.nlm.nih.gov/geo/) database with the accession number GSE3167. RMA log expression units were calculated using ‘affy’ package for the R statistical programming language. The default RMA settings were used to background correct, normalize and summarize all expression values. Second dataset was published by Sanchez-Carbayo et al., in which 81 infiltrating bladder urothelial carcinoma, 28 superficial bladder cancer, and 48 normal bladder samples were analyzed on Affymetrix U133A microarrays [50]. The gene expression level of *CSNK1D* was obtained from this study, and log2 expression level was used for statistical analysis. A 2-tailed Student’s *t*-test was then applied for the calculation of the *p* value between two different groups.

### Statistical analysis

Each experiment was performed independently with at least two biological replicates. Data in the bar graphs are presented as means ± S.D and analyzed by using the Student’s *t*-test with *p* values < 0.05 considered significant.

## Supplementary Material

Supplementary Figures
