# Supplementary material for: CK1δ as a potential therapeutic target to treat bladder cancer
Source: Aging (Albany NY). 2020 Apr 13;12(7):5764–80. doi: 10.18632/aging.102966 (PMC7185098; doi:10.18632/aging.102966)
Supplement: Supplementary Figures [file aging-12-102966-s001..pdf]

SUPPLEMENTARY FIGURES

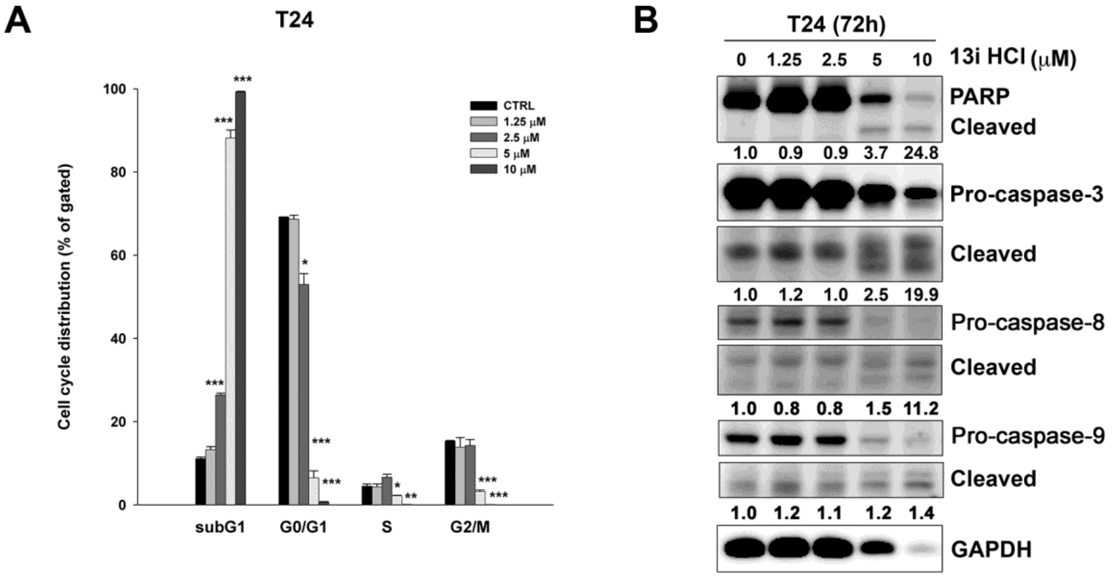

Supplementary Figure 1. Compound 13i HCl induces apoptosis in T24 cells. (**A**) T24 cells were exposed to the indicated concentrations of 13i HCl for 72 h and subjected to cell cycle analysis. Data are represented as mean ± S.D. **P*<0.05, ***P*<0.01, ****P*<0.001 compared to control cells (n=2). (**B**) T24 cells were exposed to the indicated concentrations of 13i HCl for 72 h and subjected to Western blotting with the indicated antibodies.

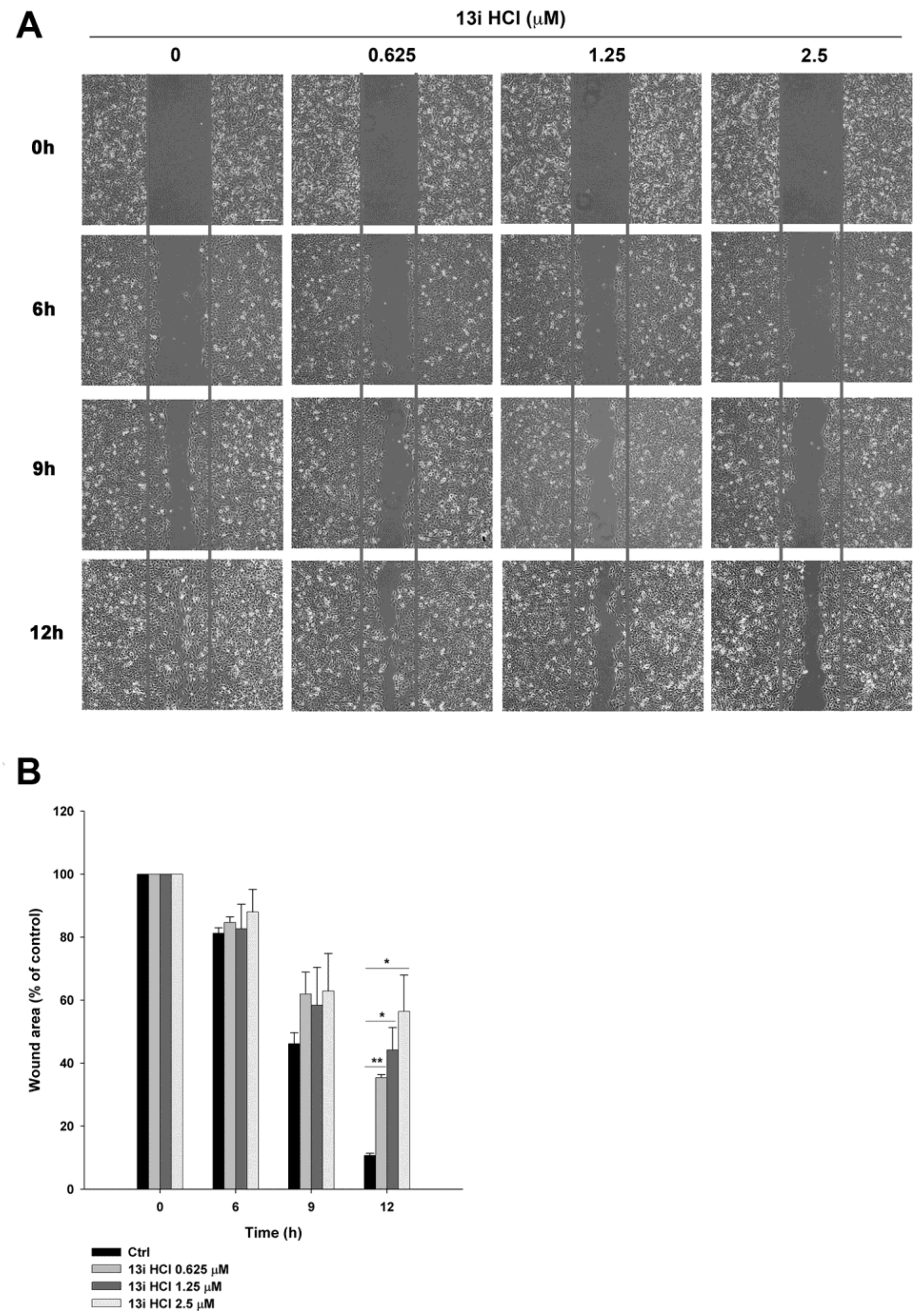

Supplementary Figure 2. Effects of 13i HCl on migratory activity in bladder cancer cells. (**A**) T24 cells were seeded into a 2-well insert on 6-well plate and allowed to attach overnight. The wound was created by removing the insert, and the cells were treated with or without 13i HCl. Images were captured at the indicated time points using an EVOS XL Core Cell Imaging System (Thermo Scientific). Scale bar = 100 mm. (**B**) Quantification of wound healing assay. Data are represented as mean ± S.D. (n=2) **P*<0.05, ***P*<0.01 compared to control cells.

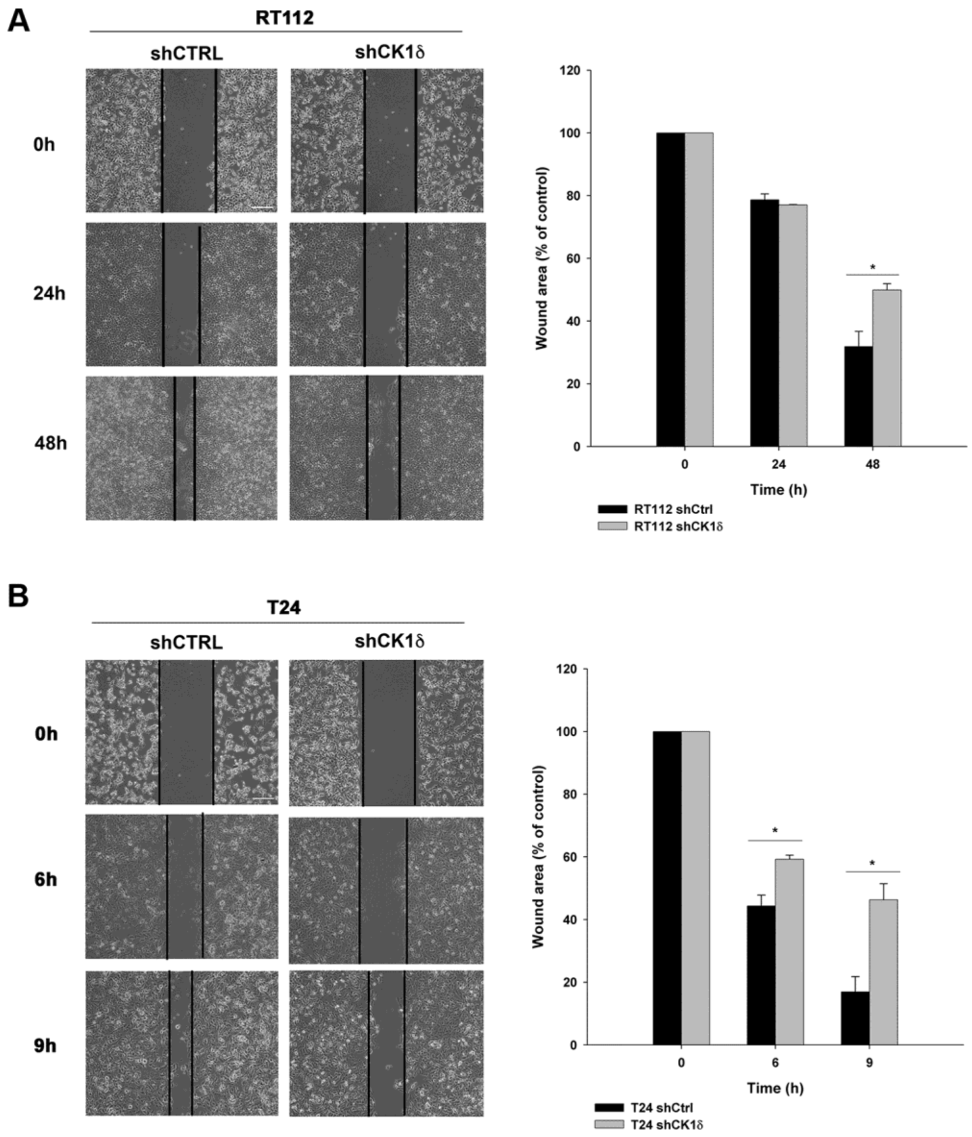

Supplementary Figure 3. Effects of CK1δ knockdown on migratory activity in bladder cancer cells. RT112 (**A**) and T24 (**B**) cells stably expressing control shRNA (shCTRL) or shCK1δ were seeded into a 2-well insert on 6-well plate and allowed to attach overnight. The wound was created by removing the insert, and the images were captured at the indicated times using an EVOS XL Core Cell Imaging System (Thermo Scientific). Scale bar = 100 mm. (**B**) Quantification of wound healing assay. Data are represented as mean ± S.D. (n=2) *P<0.05 compared to control cells.
